# More Accessible COVID-19 Treatment Through Monoclonal Antibody Infusion in the Emergency Department

**DOI:** 10.5811/westjem.2022.5.55234

**Published:** 2022-08-19

**Authors:** Sara W. Heinert, Jonathan McCoy, Pamela Ohman Strickland, Renee Riggs, Robert Eisenstein

**Affiliations:** * Rutgers Robert Wood Johnson Medical School, Department of Emergency Medicine, New Brunswick, New Jersey; † Rutgers School of Public Health, Department of Biostatistics and Epidemiology, Piscataway, New Jersey

## Abstract

**Introduction:**

Monoclonal antibody (MAB) infusion is the first treatment to manage coronavirus 2019 (COVID-19) in an outpatient setting. Yet increased risk of severe COVID-19 illness may occur from inequities in social determinants of health including access to quality healthcare. Given the safety-net nature of emergency departments (ED), a model that puts them at the center of MAB infusion may better reach underserved patients than models that require physician referral and distribute MAB at outpatient infusion centers. We examined characteristics of two groups of patients who received MAB infusion in the Robert Wood Johnson University Hospital (RWJUH) ED in New Brunswick, New Jersey: 1) patients who tested positive for COVID-19 in the ED and received ED infusion; and 2) patients who tested positive elsewhere and were referred to the ED for infusion. The process for the latter group was similar to the more common national model of patients testing COVID-19 positive in the community and then being referred to an infusion center for MAB therapy.

**Methods:**

We performed a cross-sectional retrospective health record review of all adult patients presenting to the ED from November 20, 2020–March 15, 2021 who received MAB infusion at RWJUH ED (N = 486). Patients were identified through the electronic health record system by an administrative query, with manual chart review for any additional characteristics not available through the query. We compared the two groups using chi-squared tests for categorical variables and t-tests for continuous variables.

**Results:**

We found higher proportions of Black (18% vs 6% P < 0.001, statistically significant), Hispanic (19% vs 11% P = 0.02), Medicaid (12% vs 9% P = 0.01), and uninsured (17% vs 8% P = 0.01) patients who tested positive for COVID-19 in their ED visit and then received MAB therapy during their visit than patients tested elsewhere in the community and referred to the ED for MAB therapy.

**Conclusion:**

These findings suggest that providing MAB infusion in the ED allows increased access for patients traditionally marginalized from the healthcare system, who may be at risk of longer disease duration and complications from COVID-19.

## INTRODUCTION

On November 9, 2020, the US Food and Drug Administration (FDA) issued an emergency use authorization (EUA) for the investigational monoclonal antibody (MAB) therapeutic bamlanivimab for the treatment of mild-to-moderate coronavirus 2019 (COVID-19) in adult and pediatric patients.^1^ On November 21, 2020, the FDA issued an EUA for casirivimab + imdevimab to be administered together for the treatment of mild-to-moderate COVID-19 in adult and pediatric patients.^2^ Both treatments, bamlanivimab and casirivimab + imdevimab, are monoclonal antibodies, which are laboratory-made proteins that mimic the immune system’s response to fight off harmful pathogens such as viruses^1^,^2^ and provided the first treatment to manage COVID-19 in an outpatient setting.

Both MAB therapies were authorized for patients aged 12 years or older weighing at least 40 kilograms, with positive results of direct severe acute respiratory syndrome coronavirus-2 viral testing, and who are at high risk for progression to severe COVID-19 (including those who are 65 years or older or who have certain chronic medical conditions)^1^,^2^ and who present for treatment within 10 days of developing COVID-19 symptoms. The therapies were not authorized for patients who are hospitalized or have a new oxygen requirement due to COVID-19.^1^,^2^

The risk of COVID-19 cases, hospitalizations, and deaths for racial and ethnic minority groups are higher than White, non-Hispanic persons. 3 Studies have found that racial and ethnic minority groups are more likely to have increased COVID-19 disease severity upon hospital admission compared to non-Hispanic White patients.^4^,^5^ Increased risk of severe COVID-19 illness may occur from inequities in social determinants of health including health, social, and economic inequities. 6 Thus, the US Centers for Disease Control and Prevention (CDC) recommends systems and policies that overcome obstacles to health and healthcare to help achieve health equity. 7

Within 10 days of the first EUA, the Robert Wood Johnson Barnabas Health (RWJBH) system began treating eligible patients in the ED.^8^ Other large health systems have provided MAB therapy in outpatient infusion centers^9^; however, RWJBH chose to deliver the treatment in its 11 emergency departments (ED) across New Jersey. The system has two pathways for patients to receive MAB treatment. First, patients who present to the ED and test positive for COVID-19 in the ED can be assessed for eligibility and receive MAB during the same visit. Second, patients who test positive for COVID-19 in the community and are candidates for MAB can be referred by their doctor to the ED for treatment. In this case, the referring physician usually called the ED before referring so the ED staff knew the patient was coming. Since patients were known COVID-19 positive, their care was expedited. They were quickly moved to a room where they were screened to ensure they did not need admission and MAB was ordered. In our health system, all the EDs and no infusion centers provided MAB therapy.

Given the safety-net nature of EDs, a model that puts EDs at the center of MAB infusion may better reach underserved patients than models that require physician referral and distribute MAB at outpatient infusion centers. Many underserved patients who present to the ED lack access to primary care and do not otherwise interact with the healthcare system.^10^ Thus, the characteristics of patients who test positive for COVID-19 in their ED visit and then receive MAB therapy during their visit may be different than patients who accessed testing elsewhere in the community and were referred to the ED for MAB therapy. The process for the latter group was similar to the more common national model of patients testing COVID-19 positive in the community and then being referred to an infusion center for MAB therapy.

The purpose of this study was to explore characteristics of two groups of patients who received MAB infusion in the RWJUH ED in New Brunswick, NJ: 1) patients who tested positive for COVID-19 in the ED and received infusion; and 2) patients who tested positive elsewhere and came to the ED for infusion.

## METHODS

### Study Setting

The RWJUH’s ED is a Level I trauma center that treats approximately 71,000 adult (21+ years) patients annually. The ED serves a socioeconomic and ethnically diverse patient population of approximately 24% Hispanic, 21% non-Hispanic Black, 37% non-Hispanic White, 7% Asian, and 10% other race/ethnicity (remaining <2% is unknown race/ethnicity). The population of Middlesex County, where the hospital is located, is 22% Hispanic, 12% Black, 42% non-Hispanic White, 25% Asian, and 1% other race/ethnicity.^11^ In the county, 9% of persons under 65 years old are without health insurance and 7% live in poverty.^12^

### Data Collection

We performed a cross-sectional, retrospective health record review of all adult patients presenting to the ED from November 20, 2020–March 15, 2021 who received MAB infusion at RWJUH ED (N = 486). Patients were identified through the electronic health record system AllScripts Sunrise Clinical Manager (Practice EHR, Plano, TX) by an administrative query, with manual chart review for any additional characteristics not available through the query, such as comorbidities. For data entry, the study team created a standardized data collection form using REDCap electronic data capture tools hosted at Rutgers University.^12^,^13^ REDCap (Research Electronic Data Capture) is a secure, web-based software platform designed to support data capture for research studies, providing 1) an intuitive interface for validated data capture; 2) audit trails for tracking data manipulation and export procedures; 3) automated export procedures for seamless data downloads to common statistical packages; and 4) procedures for data integration and interoperability with external sources. Data entry was frequently reviewed by the two lead investigators, and any errors were reported back to the person who had entered the data to correct them.

### Statistical Analysis

Analysis compared demographics, medical characteristics, and vital signs at triage for patients who tested COVID-19 positive in the ED and received MAB infusion to patients who tested COVID-19 positive elsewhere and came to the ED for infusion. We used chi-squared tests for categorical variables and t-tests for continuous variables using Stata version 16.0 (StataCorp LLC, College Station, TX). We carried out an analysis for the statistical significance of the results using the Bonferroni correction for multiple variables. This study was approved by the Rutgers University Institutional Review Board.

## RESULTS

A total of 819 patients tested COVID-19 positive and/or received MAB in the ED, of whom 486 received MAB in the ED. Three-hundred thirty-three (333) patients did not receive MAB, of whom 75 (23%) were eligible for MAB. The table shows characteristics of patients who received MAB infusion in the RWJUH ED, comparing patients who tested COVID-19 positive in the ED to those who tested positive elsewhere and were referred to the ED for infusion. Compared to patients who tested positive in the community and were referred to the ED (n = 334), patients who tested positive for COVID-19 in the ED (n = 152) were significantly different in race (*P* <0.001), ethnicity (*P* = 0.02), and insurance type (*P* = 0.01) with higher proportions of Black (18% vs 6% P < 0.001) and Hispanic (19% vs 11% P = 0.02) patients. There were higher proportions of Medicaid patients tested in the ED than outside the ED (12% vs 9% P = 0.01) and double the proportion of uninsured patients (self-pay and charity care) (17% vs 8% P = 0.01). There were no significant differences in gender between the two groups.

We also analyzed medical characteristics and vital signs at triage (not shown), of which only heart rate and systolic blood pressure (BP) were significantly different between the two groups. While mean heart rate was statistically significantly higher for patients testing positive in the ED (93.3, standard deviation [SD] = 17.3) than patients referred to the ED for infusion (89.1, SD = 16.0), this is unlikely to be of any clinical significance. Likewise, systolic BP was statistically significantly lower for patients testing positive in the ED (137.3, SD = 21.1) than patients referred to the ED for infusion (141.6, SD = 23.2). Race was the only finding that remained significant after using the Bonferroni correction (not shown).

## DISCUSSION

Overall, there were significant demographic differences but few medical differences between patients who tested COVID-19 positive in the ED and received MAB infusion compared to patients who tested COVID-19 positive in the community with referral to the ED for MAB infusion. There were significantly higher proportions of underserved (racial/ethnic minority, Medicaid, and uninsured) patients who tested positive for COVID-19 in their ED visit and then received MAB therapy during their visit than patients tested elsewhere in the community and referred to the ED for MAB therapy. Medical differences were of limited clinical significance but may highlight that patients diagnosed and treated in the ED were slightly sicker on average than the referred population. After using the Bonferroni correction for comparing multiple variables, race was the only variable that was significantly different between the two groups. However, this study was exploratory with highly correlated covariates such as race with ethnicity and insurance type, suggesting that race itself was likely not the only determining factor, and ethnicity and insurance type were statistically significant prior to Bonferroni correction.

These findings are promising for creating programs to better serve underserved patients, and some health systems that initially referred eligible ED patients to outpatient infusions centers have since shifted their model to include MAB distribution in the ED.^14^ However, it is also important to consider and plan for potential ED workflow issues that can arise from providing MAB infusion in the ED. This can include additional use of beds and staffing, which may already be in short supply, especially during a pandemic.

## LIMITATIONS

Our study had some limitations. First, this was a retrospective quantitative study of healthcare utilization. Two areas of potential bias in chart review studies are that the data in patient records is inaccurate and that the data is collected with non-systematic and potentially inaccurate methodology. Medical variables for our study were objective in nature and demographics were self-reported by the patient, which would minimize bias compared to ED staff-reported patient demographics. While several best practice methods of chart review studies^15^ were completed, such as standardized abstraction forms, there were some practices that we were unable to accomplish, specifically blinding abstractors to study hypotheses and measuring interrater reliability.

We were also unable to measure ED patients’ logistical ability to receive MAB infusion in an infusion center if the MAB was not available in the ED. Neither were we able to quantify who tested positive for COVID-19 in the community but were not able to come to the ED for the infusion, nor the demographics of patients who received MAB at infusion centers. Second, our dataset did not include primary care physician (PCP). One explanation for our findings is that if patients test positive for COVID-19 in the community and do not have a PCP, then no one is advocating for them or educating them to go to the ED for MAB infusion if they are eligible for the treatment. Thus, there may be a much higher proportion of patients with PCPs in the community who were tested and referred to the ED than those in the ED-tested group. However, we were unable to observe this difference without this variable in the study data.

## CONCLUSION

These findings suggest that providing MAB infusion in the ED allows increased access for patients traditionally marginalized from the healthcare system, who may be at risk of longer disease duration and complications from COVID-19.

## Figures and Tables

**Table 1:** Characteristics of patients receiving monoclonal antibody infusion in the emergency department (ED) comparing patients testing COVID-19 positive in the ED to patients testing COVID-19 positive elsewhere with referral for infusion to the ED.

Characteristics	Overall (N = 486) N (%)	Tested Elsewhere (n = 334) n (%)	Tested in ED (n = 152) n (%)	P-value
Demographics				
Gender				
Women	250 (51%)	165 (49%)	85 (56%)	0.18
Men	236 (49%)	169 (51%)	67 (44%)	
Race (n = 483)				
Asian	29 (6%)	22 (7%)	7 (5%)	<0.001*
Black	48 (10%)	21 (6%)	27 (18%)	
Other	168 (35%)	107 (32%)	61 (40%)	
White	238 (49%)	182 (55%)	56 (37%)	
Ethnicity (n = 476)				
Non-Hispanic	413 (87%)	294 (89%)	119 (82%)	0.02
Hispanic	63 (13%)	36 (11%)	27 (19%)	
Insurance type				
Charity	8 (2%)	2 (<1%)	6 (4%)	0.01
Medicaid	49 (10%)	31 (9%)	18 (12%)	
Medicare	147 (30%)	106 (32%)	41 (27%)	
Other	3 (1%)	1 (<1%)	2 (1%)	
Private	234 (48%)	169 (51%)	65 (43%)	
Self-Pay	45 (9%)	25 (8%)	20 (13%)	

* Only patient characteristic that was statistically significant after Bonferroni correction.

*ED*, emergency department; *COVID-19*, coronavirus disease of 2019; *P-value*, probability value.
